# Acetylated Rhamnogalacturonans from Immature Fruits of *Abelmoschus esculentus* Inhibit the Adhesion of *Helicobacter pylori* to Human Gastric Cells by Interaction with Outer Membrane Proteins

**DOI:** 10.3390/molecules200916770

**Published:** 2015-09-15

**Authors:** Christian Thöle, Simone Brandt, Niyaz Ahmed, Andreas Hensel

**Affiliations:** 1 Institute of Pharmaceutical Biology and Phytochemistry, University of Münster, Münster 48149, Germany; E-Mails: c.thoele@uni-muenster.de (C.T.); simone.brandt@uni-muenster.de (S.B.); 2 Pathogen Biology Laboratory, Department of Biotechnology and Bioinformatics, University of Hyderabad, Hyderabad, Telangana 500046, India; E-Mail: ahmed.nizi@gmail.com

**Keywords:** *Abelmoschus esculentus*, acetylation, adhesion, antiadhesion, BabA, *Helicobacter pylori*, okra, rhamnogalacturonan

## Abstract

Polysaccharide containing extracts from immature fruits of okra (*Abelmoschus esculentus*) are known to exhibit antiadhesive effects against bacterial adhesion of *Helicobacter pylori* (*H. pylori*) to stomach tissue. The present study investigates structural and functional features of polymers responsible for this inhibition of bacterial attachment to host cells. Ammonium sulfate precipitation of an aqueous extract yielded two fractions at 60% and 90% saturation with significant antiadhesive effects against *H. pylori*, strain J99, (FE_60%_ 68% ± 15%; FE_90%_ 75% ± 11% inhibition rates) after preincubation of the bacteria at 1 mg/mL. Sequential extraction of okra fruits yielded hot buffer soluble solids (HBSS) with dose dependent antiadhesive effects against strain J99 and three clinical isolates. Preincubation of *H. pylori* with HBSS (1 mg/mL) led to reduced binding to 3ʹ-sialyl lactose, sialylated Le^a^ and Le^x^. A reduction of bacterial binding to ligands complementary to BabA and SabA was observed when bacteria were pretreated with FE_90%_. Structural analysis of the antiadhesive polysaccharides (molecular weight, monomer composition, linkage analysis, stereochemistry, and acetylation) indicated the presence of acetylated rhamnogalacturonan-I polymers, decorated with short galactose side chains. Deacetylation of HBSS and FE_90%_ resulted in loss of the antiadhesive activity, indicating esterification being a prerequisite for antiadhesive activity.

## 1. Introduction

The immature fruits of *Abelmoschus esculentus* (*A. esculentus*) (L.) Moench, Malvaceae, also known as okra or lady’s finger, are widely used as a food vegetable in Asia, Africa, and South America. Because of its high amount of polysaccharides, which have been characterized as pectin-like mucilages [1], okra is also used in traditional medicine as a dietary meal to treat gastric irritation. Aqueous extracts of okra fruits have been described to possess antiadhesive properties against the bacterial adhesion of *Helicobacter pylori* (*H. pylori*) to human stomach tissue [2]. The antiadhesive effects of the crude extract are caused by an interaction with the bacteria, while pretreatment of human stomach tissue with the plant polysaccharides prior to the addition of *H. pylori* does not lead to reduced bacterial adhesion [1,2]. The extract has no direct cytotoxic effects against *H. pylori* [1,2] and does not influence the gene expression of the main bacterial virulence factors [2]. Despite the fact that the aqueous extract inhibits the adhesive binding of the membrane proteins BabA, SabA and HpA to its specific ligands, it has been shown that radiolabeled ligands of this extract bind non-specifically to membrane structures in the vicinity of the bacterial adhesins, but do not necessarily interact directly with BabA/SabA [2]. This lead to the conclusion that non-specific, charge-dependent interactions between high molecular compounds from Okra fruits and the *H. pylori* surface lead to strong antiadhesive effects under *in vitro* conditions.

As in all previous studies the aqueous fresh extract from immature okra fruits has been used for functional investigations, the following study aimed the isolation and characterization towards the fractionation of the high molecular compounds of the extract and pinpointing the lead structures responsible for the antiadhesive effect against the attachment of *H. pylori*. As shown by the following investigation, esterified pectin-like rhamnogalacturonan I polymers are responsible for the antiadhesive effects; interestingly, the antiadhesive activity was shown to be dependent on the esterification.

## 2. Results and Discussion

### 2.1. Polysaccharide Isolation from A. esculentus

**Figure 1:**
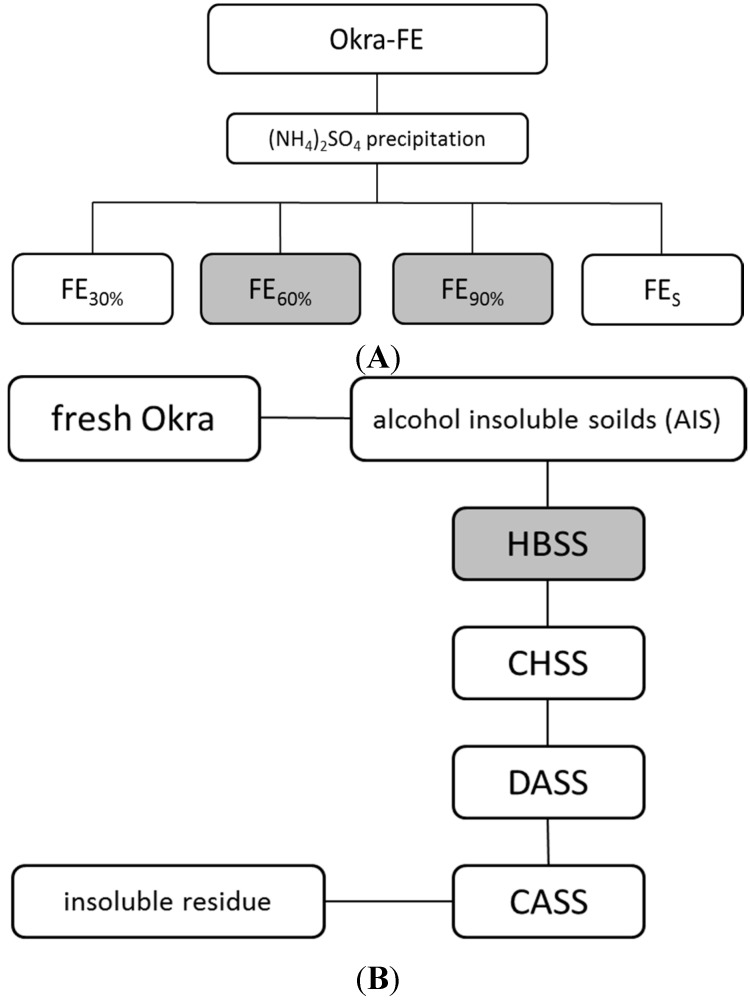
Extraction schemes of different okra pulp extracts. (**A**) Purification of Okra fresh extract (Okra-FE) [2] by ammonium sulfate precipitation with saturation levels of 30%, 60%, and 90%; (**B**) Stepwise extraction of Okra polysaccharides according to [3]. Fractions with grey background were chosen for further structural characterization due to best antiadhesive effects.

Fresh immature fruits of *A. esculentus* were separated into pulps (66%), seeds (17%), and calyx. The aqueous extract Okra-FE [2] obtained from the pulp material was directly subjected to ammonium sulfate precipitation (Figure 1A). At saturation levels of 30% (FE_30%_), 60% (FE_60%_), and 90% (FE_90%_), high molecular material was obtained after ammonium sulfate precipitation with yields of 0.05%, 0.06%, and 0.14%, respectively, referred to the fresh weight of the pulp material. The remaining supernatant (FE_S_) corresponds to 0.12% of fresh pulps.

In a second approach, okra polysaccharides were extracted sequentially with different solvents [3] (Figure 1B). After removal of lipophilic and low molecular weight compounds, in a first extraction step, a pectin-rich fraction was obtained from the alcohol insoluble solids (AIS), in the following named as hot buffer soluble solids (HBSS); by extraction with EDTA-containing solvent, polysaccharides were obtained named in the following as chelating agent soluble solids (CHSS) [4]. By use of alkaline extractans xyloglucan-like, polymers were obtained in the fractions named as diluted alkaline soluble solids (DASS) and concentrated alkali soluble solids (CASS) [3]. The respective chemical composition and analytical characterization of these polysaccharides has recently been described in detail, indicating that polymers from HBSS consist mainly of polysaccharides with a rhamnogalacturonan I structure with short galactan side chains, whereas the alkaline fraction CASS contains mainly xyloglucan [3,4].

### 2.2. Antiadhesive Activity of Okra Polysaccharides against H. pylori

**Figure 2:**
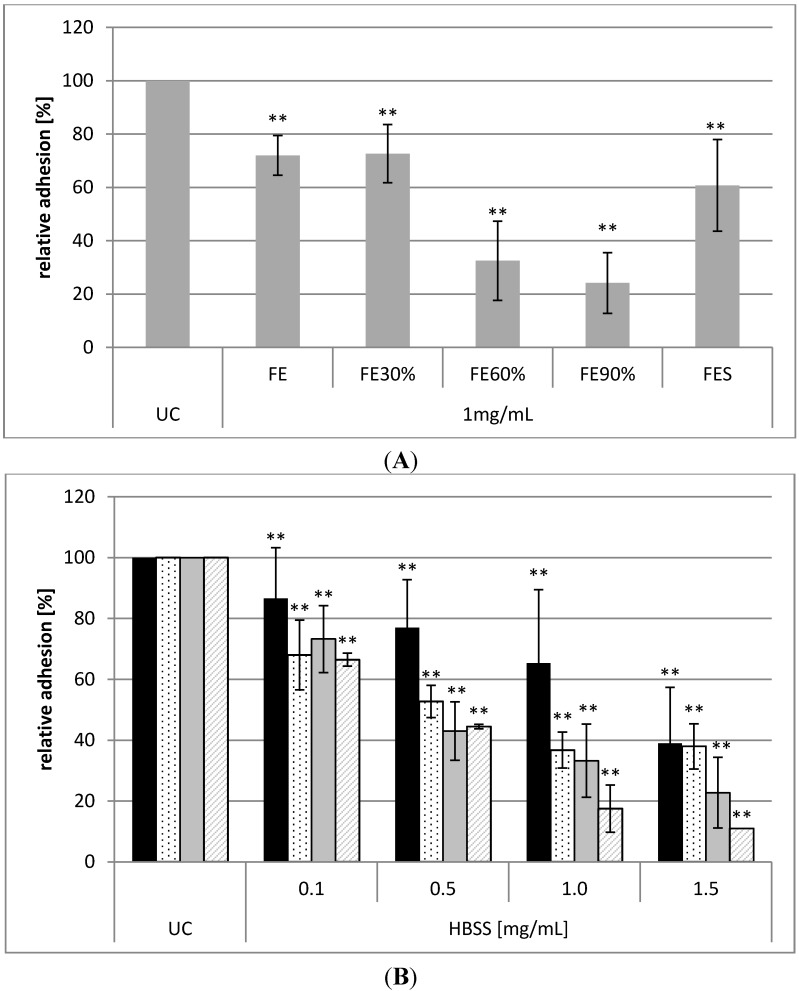
Relative adhesion (%, related to the untreated control UC) of FITC-labeled *H. pylori* to AGS cells (**A**) after pretreatment of the bacteria (strain J99) with 1 mg/mL of different fractions of purified Okra-FE; (**B**) Relative adhesion of different *H. pylori* strains (black: J99, dotted: P12, grey: 26695, dashed: TN-2) after pretreatment with 1 mg/mL HBSS. Values are mean ± SD of three independent experiments with two technical replicates; ** *p* < 0.01 related to the untreated control UC (=100%).

For investigation of antiadhesive activities of Okra polysaccharides against *H. pylori*, an *in vitro* flow cytometric assay with human gastric epithelial AGS cells and FITC-labeled bacteria was used to quantify potential antiadhesive effects [5]. As described recently [2], the aqueous extract Okra-FE inhibits the bacterial adhesion to AGS cells significantly in a concentration dependent manner with inhibition rates of bacterial adhesion of about 30% at a concentration of 1 mg/mL. For pinpointing the relevant compounds responsible for this antiadhesive effect, fractionation of Okra-FE by ammonium sulfate precipitation yielded three subfractions. Functional testing against the adhesion of *H. pylori* indicated a basic inhibitory activity for FE_30%_ (27% ± 10% inhibition) and high blocking rates for FE_60%_ (68% ± 15%) and especially for FE_90%_ (75% ± 11%) after preincubation of bacteria at 1 mg/mL of the respective polysaccharides (Figure 2A). Surprisingly, the non-precipitable supernatant of the extract after ammonium sulfate precipitation also still contains molecules interacting with *H. pylori*, resulting in about 40% ± 17% inhibition of bacterial adhesion.

Comparing the four different polysaccharide fractions, HBSS, CHSS, DASS, and CASS, obtained from the sequential extraction protocol [3], all extracts showed an antiadhesive effect in a concentration dependent manner against strain J99 (data not shown). HBSS and CASS exhibited best antiadhesive effect with, respectively, 69% ± 17% and 53% ± 8% inhibition of bacterial adhesion after preincubation at 2 mg/mL. At the same concentration a preincubation with CHSS and DASS decreased the adhesion of *H. pylori* to AGS cells to 48% ± 16% and 15% ± 14%, respectively. To exclude strain specific effects the quantitative antiadhesion assay was additionally performed with the *H. pylori* strains and clinical isolates P12, 26695, and TN-2, beside the lab strain J99. As highlighted in Figure 2B, HBSS was significantly active against all four *H. pylori* strains in a dose dependent manner, but to a different extent (Figure 2B).

All fractions tested showed no cytotoxic effect against *H. pylori*, which was proven by agar diffusion assay in the concentration range from 0.5 to 1.5 mg/mL. In addition, no cytotoxic effect against the AGS host cells was observed by determination of mitochondrial activity by MTT assay.

### 2.3. Analytical Characterization of Okra Polysaccharides

**Figure 3:**
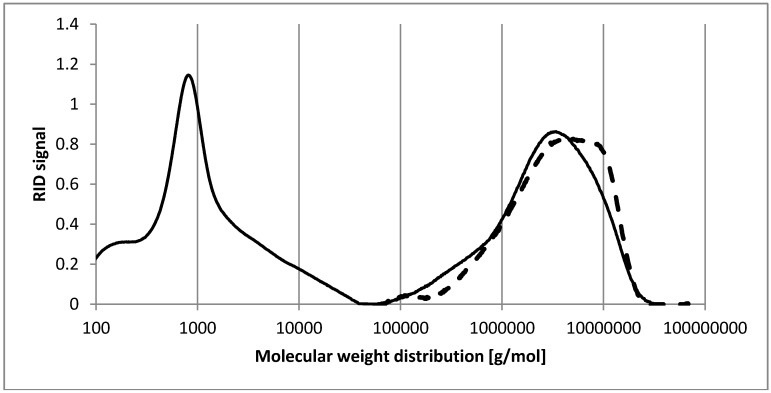
Molecular weight distribution of FE_60%_ (black line) and FE_90%_ (dashed line) determined by HP-SEC, according to standard calibration with pullulans

The highly active fractions FE_60%_, FE_90%_, and HBSS were chosen for detailed structural analysis. High performance size exclusion chromatography (HP-SEC) was used to determine the molecular weight (Figure 3). The fraction FE_60%_ consisted of two different polymers. By standard calibration with pullulans, the average molecular weight of these polysaccharides was determined with 10 kDa and 2800 kDa, respectively. In contrast, FE_90%_ consists of only a single polymer of 4600 kDa.

The average molecular weight of a single peak polymer in HBSS was calculated with 680 kDa (data not shown).

The protein content in FE_60%_ (38%) was shown to be higher than in FE_90%_ (19%) and HBSS (15%), whereas HBSS and FE_90%_ contain both 15% uronic acids and FE_60%_ only 4%.

Standard methods for carbohydrate analysis were performed as follows: the monosaccharide composition was determined by High-performance anion-exchange chromatography with pulsed amperometric detection (HPAEC-PAD) after TFA hydrolysis of native polysaccharides. Quantification was done by external calibration with reference monosaccharide standard solutions. Results obtained were confirmed by GC-MS determination of alditol acetates obtained after reduction and acetylation of the TFA-hydrolyzed polymers. Linkage analysis was carried out via methylation analysis after reductive deuteration of uronic acids. The partially methylated alditol acetates (PMAA) were analyzed by GC-MS. Table 1 summarizes the analytical composition of fractions FE_60%_, FE_90%_, and HBSS.

**Table 1:** Analytical composition (mol %) of different Okra pulp polysaccharides with highest antiadhesive properties against *H. pylori*: monosaccharide composition as determined by HPAEC-PAD after TFA hydrolysis, results of linkage analysis after methylation of carboxyl-reduced polymers and GC-MS identification of PMAAs. tr.: traces.

	Carbohydrate	Linkage-Type	Amount GC-MS (mol %)		
			FE_60%_	FE_90%_	HBSS
Protein			37.7	18.8	15.1
Uronic acids		total	4.0	16.0	16.2
	d-galacturonic acid	1,4-Gal*p*	3.7	15.0	14.7
	d-glucuronic acid	1,4-Glc*p*A	0.3	1.0	1.5
Neutral sugars	d-glucose	total Glc	10.3	5.8	26.8
		1-Glc*p*	5.9	3.6	2.3
		1,4-Glc*p*	4.4	1.2	19.9
		1,6-Glc*p*			1.8
		1,2,4-Glc*p*		0.9	2.1
		1,4,6-Glc*p*			0.7
	d-galactose	total Gal	27.0	33.5	26.8
		1-Gal*p*	10.7	11.0	13.3
		1,4-Gal*p*	13.2	18.5	2.1
		1,2-Gal*p*	1.4	1.9	3.5
		1,6-Gal*p*	1.2	1.5	
		1,2,4-Gal*p*			1.1
		1,3,6-Gal*p*	0.5	0.6	2.8
		1,4,6-Gal*p*			4.1
	l-rhamnose	total Rha	16.8	21.5	13.7
		1,2-Rha*p*	1.3	1.6	0.4
		1,2,4-Rha*p*	15.5	19.9	13.3
	d-mannose	total Man	2.2	1.2	0.1
		1,4-Man*p*			0.1
		1,4,6-Man*p*			tr.
	d-xylose	total Xyl	0.4	0.4	0.1
		1-Xyl*p*	tr.		tr.
		1,2-Xyl*p*	0.4	0.4	0.1
	d-arabinose	total Ara	0.7	1.1	1.4
		1-Ara*f*	0.1	0.1	0.5
		1,5-Ara*f*	0.6	1.0	0.9
	d-fucose	total Fuc	0.9	1.6	

**Figure 4:**
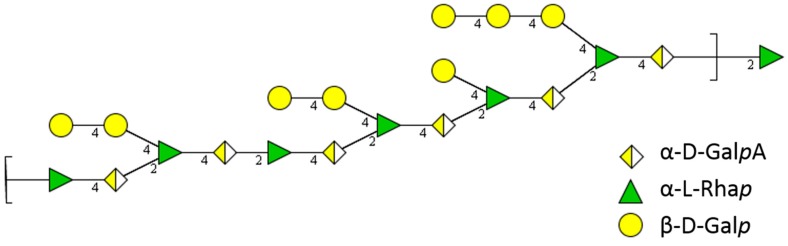
General structural elements of rhamnogalacturonan I unit present in HBSS, FE_60%_, and FE_90%_. Numbers refer to linkage position of previous sugar to C-1 of next carbohydrate. Structure designed with GlycoWorkbench software, V. 2.1 [6].

All three fractions mainly consist of galactose, rhamnose and galacturonic acid. The relation of the three sugars indicates the presence of a rhamnogalacturonan I backbone consisting of repeating units of 1,4-d-galacturonic acid, linked to 1,2-l-rhamnose (Figure 4). At position 4, the rhamnose units are mainly linked to short galactan side chains of one or two galactose moieties. These findings are in coincidence with previous studies [4]. Other monosaccharides like arabinose, xylose or mannose are only present in minor percentage or only in traces.

**Figure 5:**
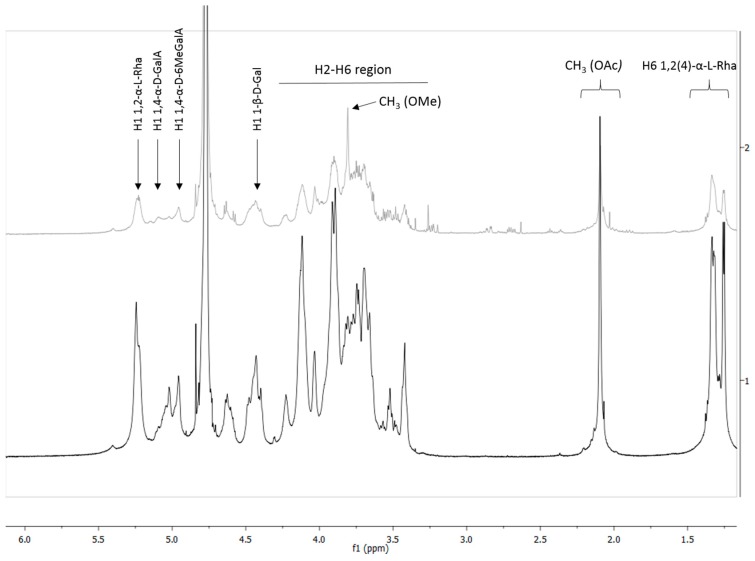
Overlay of ^1^H-NMR spectra of HBSS-1 (**grey**) and FE_90%_-1 (**black**), recorded at 600 MHz.

**Figure 6:**
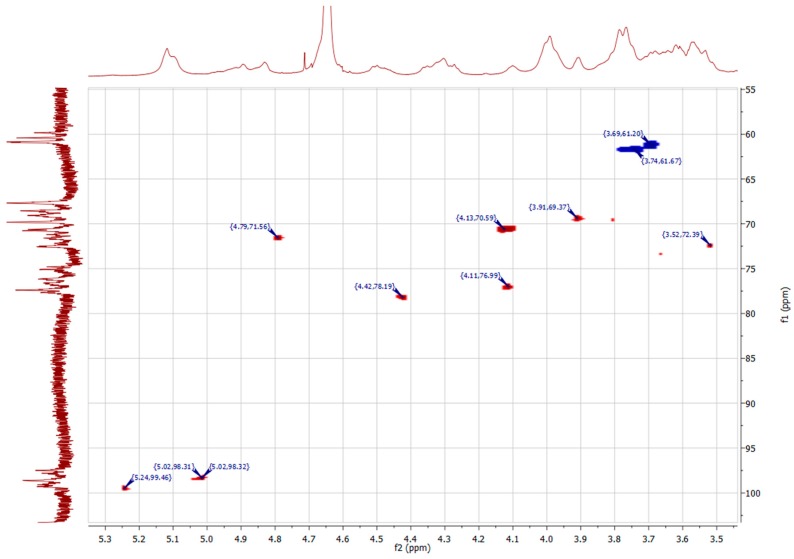
Relevant details of ^1^H-^13^C HSQC NMR spectra of FE_90%_-1. Spots with highest signal intensity are labeled with chemical shifts (δ, ppm).

To gain information about the anomeric constitution of respective monosaccharides, 1D- and 2D-NMR spectra were recorded. Since the high molecular weight of native polymers limits the solubility in NMR solvents, fractions HBSS and FE_90%_ were subjected to a mild TFA hydrolysis. The hydrolysates were purified by gel chromatography on Sepharose^®^ CL-6B. Fractions containing polysaccharides with a molecular weight ranging from 5 kDa–50 kDa and >50 kDa were combined and named in the following as HBSS-2/FE_90%_-2 and HBSS-1/FE_90%_-1, respectively. ^1^H- and ^13^C-NMR spectra of all fractions were recorded at 600 MHz and 150 MHz, respectively. The spectra of HBSS-1 and FE_90%_-1 had a better resolution than that of the respective lower molecular weight fraction, probably due to higher sample amount used for analysis. Nevertheless, no major differences in the NMR spectra of low and high molecular weight fractions were observed. A comparison of ^1^H-NMR spectra of HBSS-1 and FE_90%_-1 (Figure 5) shows a high analogy between both spectra. The signals at 1.25 and 1.35 ppm correspond to H-6 of 1,2-α-l-rhamnose and 1,2,4-α-l-rhamnose, respectively. The presence of two doublets at 1.21 and 1.35 ppm indicates the presence of rhamnogalacturonan I domains [7,8]. The signal at 2.10 ppm proves the presence of *O*-acetyl substituent [3], whereas the signal intensity is clearly higher for FE_90%_-1 than for HBSS-1. It seems interesting that HBSS-1 has a lower degree of acetylation compared to FE_90%_. This correlates again well with the stronger antiadhesive activity of FE_90%_ compared to HBSS (see Figure 2). On the contrary, the signal for methyl esters at 3.82 ppm [8] is higher in HBSS-1 than in FE_90%_-1 indicating a higher content of methyl esters. Therefore, it can be concluded that the acetylation seems to have a higher impact on the antiadhesive properties than methylation. By comparing the 1D- and 2D-NMR data (Figure 5 and Figure 6; Table 2) with recently published data [3,7,8], the presence of t-β-d-Gal*p*, 1,4-β-d-Gal*p*, 1,2,(4)-α-l-Rha*p*, and 1,4-α-d-Gal*p*A was proven, which is in accordance with the data obtained from methylation analysis (Table 1). These findings underline the proposed structure of repeating RG-I units within the backbone with galactan side chains as the main polysaccharides in Okra pulps.

**Table 2:** Chemical shifts of main glycosyl residues of FE_90%_-1 from ^1^H-, ^13^C-NMR and ^1^H-^13^C-HSQC.

Glycosyl-Residue	Chemical Shifts, δ (ppm)					
	H1/C1	H2/C2	H3/C3	H4/C4	H5/C5	H6/C6
→ 2)-α-l-Rha*p*-(1 →	5.24/99.46	3.52/72.39	3.89/-	3.44/-	3.79/-	1.25/17.25
→ 2,4)-α-l-Rha*p*-(1 →	5.23/99.46	-	3.93/-	-	3.91/69.37	1.32/17.57
→ 4)-α-d-Gal*p*A-(1 →	5.02/98.31	4.79/71.56	4.11/76.99	4.42/78.19	-	175.41
→ 4)-α-d-Gal*p*A-OAc-(1 →	5.02/98.32	4.13/70.59	-	-	-	
→ 4)-α-d-6MeGal*p*A-(1 →	4.96/100.80	3.76/-	4.01/-	-	-	174.04
→ 4)-β-d-Gal*p*-(1 →	4.05/69.24	-	3.74/61.67	-	3.71/70.45	3.70/65.40
β-d-Gal*p*-(1 →	4.45/-	3.42/-	3.67/-	-	-	-
-OAc	2.09/21.25					
-OMe	3.82/53.70					

-: not determined.

### 2.4. Influence of Esterification on Antiadhesive Activity

**Figure 7:**
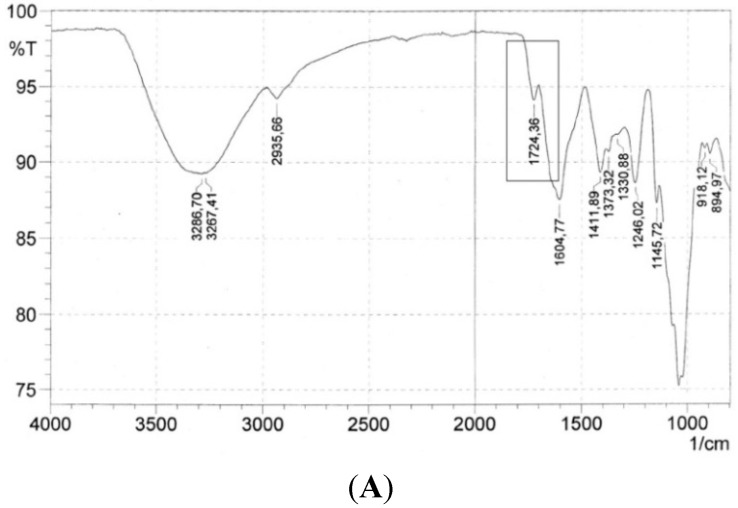

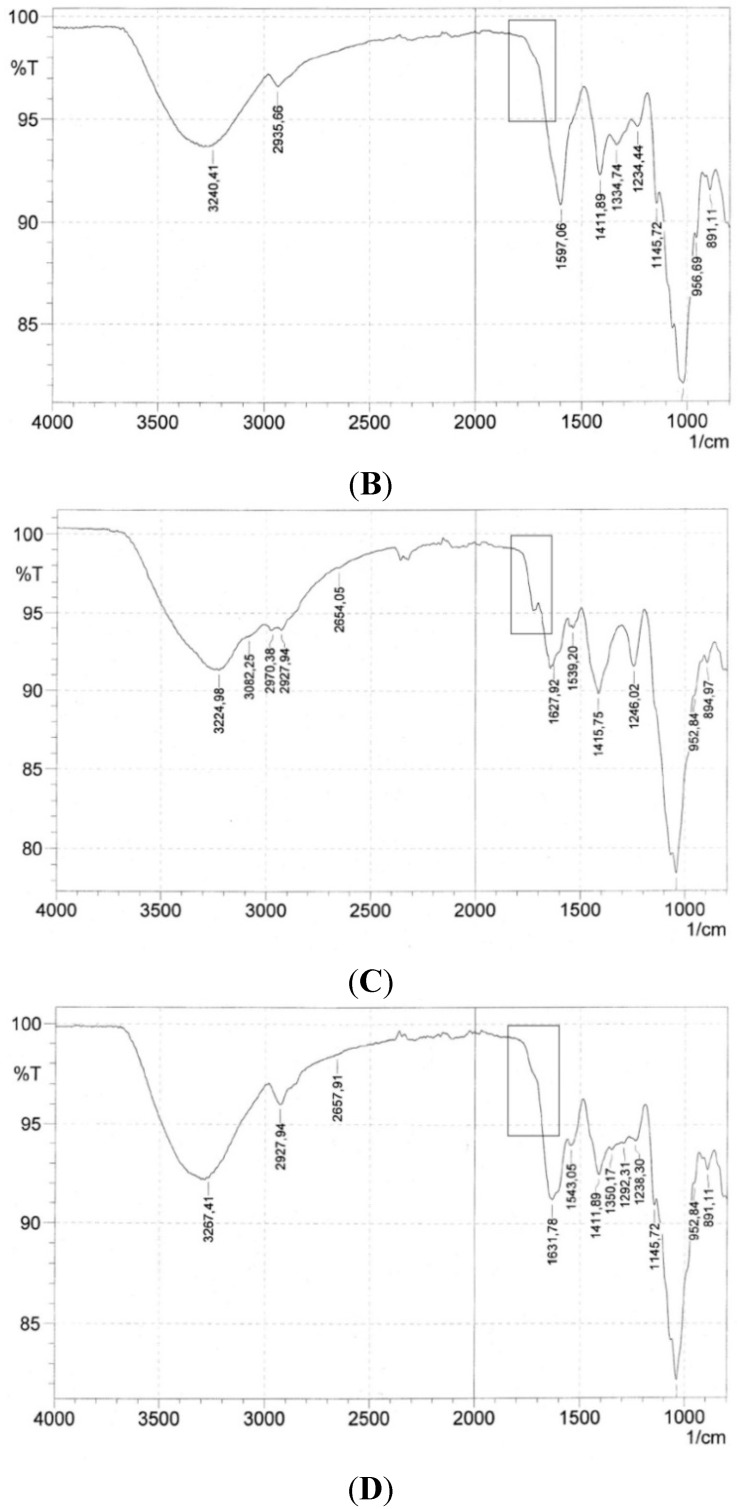
FT-IR spectra of native (**A**); and saponified (**B**); HBSS and native (**C**); and saponified (**D**) FE_90%_ polymers with antiadhesive activity against *H. pylori*. Labeled area shows signals of methyl- and acetylesters.

Pectins and pectin-like molecules are known to be esterified with either MeOH at the C_6_-carboxyl group of the uronic acids or with acetic acid at hydroxyl groups. In many cases, the degree of esterification (DE) can be directly related to the physicochemical and functional properties of such polymers [9]. Since the analytical investigation of the bioactive Okra polysaccharides indicated the presence of RG I-polymers, the presence of esters was also assumed. For this reason, Fourier transformation-infrared (FT-IR) spectra of the three respective polymers were recorded, using pectin samples with a known DE as reference. As shown in Figure 7 IR spectra of all three polysaccharides had a high absorbance between wavenumbers of 950 and 1300 cm^−1^. This region is claimed as the “fingerprint” region, which is specific for each carbohydrate. Due to complex interacting vibrational modes, it is quite difficult to assign bands to a specific atom vibration group [10]. The native polymers of HBSS and FE_90%_ show a small band at 1740 cm^−1^, which can be related to C=O stretching vibration of methyl esterified to acetyl esters [10]. This signal seems to be usable for determination of esterification and the intensity of this band correlated also directly with the DE of the reference pectins (data not shown). Herewith, the presence of esterified RG-I structures was proven.

**Figure 8:**
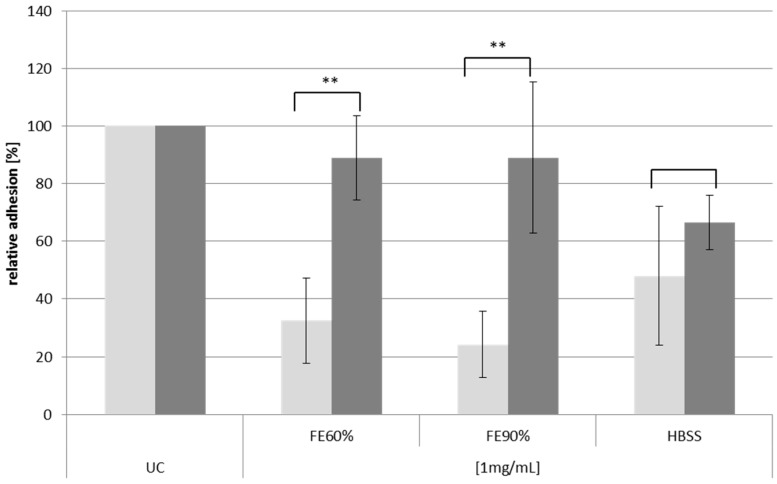
Relative adhesion of FITC-labeled *H. pylori* on AGS cells after pretreatment with 1 mg/mL of native and saponified polymers from Okra pulp. Light bars: native fractions, dark bars: saponified fractions. UC: untreated control (=100%), ** *p* < 0.01.

In contrast, a high DE might have a drastic influence on the steric and physicochemical properties of pectins and could also influence enzymatic degradation of the polysaccharide backbone and side chains [3] or influence the molecular interaction with protein targets. To investigate the influence of esterification on the antiadhesive activity of the Okra polysaccharide against *H. pylori* alkaline saponification of the polymers was performed by incubating the samples in diluted sodium hydroxide and using NaBH_4_ to prevent unspecific polysaccharide stripping [3]. Deesterified polysaccharides were obtained in recovery yields of 77%, 72%, and 64% for FE_60%_, FE_90%_, and HBSS, respectively. As expected, the relevant ester signal in the FT-IR spectra after saponification was not detectable any more (Figure 7B,D). A comparison of the monosaccharide and uronic acid composition of both native and saponified samples revealed no major differences. Also the protein content of the three samples did not change significantly (data not shown). This leads to the assumption that the polymers are not degraded or altered unspecifically during the saponification procedure, except that they are de-esterified. Surprisingly, within functional testing, the saponified polymers did show strongly reduced antiadhesive activity for FE_60%_ and FE_90%_ (Figure 8). The activity of FE_60%_ and FE_90%_ was almost completely lost, while for HBSS only a small, but not significantly decrease in the antiadhesive properties was observed. Therefore, the presence of acetyl- and methyl esters seems to be a prerequisite for antiadhesive activity of the rhamnogalacturonans.

### 2.5. Interaction of Okra Polysaccharide with H. pylori Adhesins: Dot Blot Overlay Assay

For investigation of the molecular targets of okra polysaccharides to *H. pylori* outer membrane proteins, a semiquantitative dot blot overlay assay [2,11] was performed to pinpoint the respective bacterial adhesins blocked by the antiadhesive polymers. Therefore, putative ligands in form of the respective neoglycoproteins known to interact specifically with *H. pylori* adhesins were immobilized by spotting on PVDF membranes. A representative selection of ligands identified for *H. pylori* adhesins used for these experiments were: Le^b^- and H type I-conjugates (which interact specifically with BabA); sialyl-Lewis^a^ and laminin (known for interacting with SabA); and fibronectin (with a not-yet-determined bacterial adhesin affinity). In addition to the use of human serum albumin (HSA) and bovine serum albumin (BSA) as controls to exclude non-specific binding of *H. pylori* to spotted compounds on the membrane, 6ʹ-sialyllactose also served as a control.

**Figure 9:**
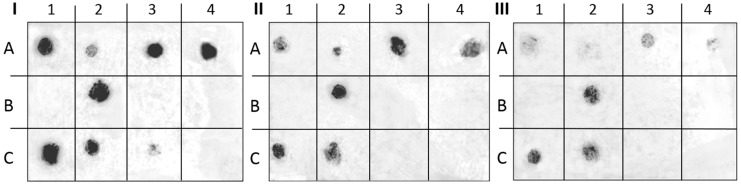
Representative images of the adhesion of FITC-labeled *H. pylori* strain J99 to immobilized ligands within dot blot overlay assay on PVDF membranes: (**I**) untreated control; and pretreated bacteria with (**II**) HBSS (1 mg/mL); and (**III**) FE_90%_ (1 mg/mL). (Neo)glycoproteins spotted on PVDF membranes (1 μg per spot) were overlaid with FITC-labeled *H. pylori* and adherent bacteria were detected by fluorescence imaging. The respective locations of spotted (neo)glycoproteins are indicated below.

The untreated FITC-labeled *H. pylori* are binding to the immobilized sialyl-Lewis^a^ and sialyl-Lewis^x^ as ligands of the adhesin SabA as well as to H type I for BabA. A strong binding to spotted fibronectin, laminin, and lactoferrin was observed (Figure 9).

**Table:** 

**A 1:** Sialyl-Lewis^a^-HSA	**A 2:** Sialyl-Lewis^x^-HSA	**A 3:** Laminin	**A 4:** Lactoferrin
**B 1:** Lewis^b^-HSA	**B 2:** H type I-HSA	**B 3:** HSA	**B 4:** BSA
**C 1:** Fibronectin	**C 2:** 3ʹ-Sialyllactose-HSA	**C 3:** 6ʹ-Sialyllactose-HSA	**C 4:** Fetuin

A preincubation of *H. pylori* with HBSS (1 mg/mL) leads to reduced binding to 3ʹ-sialyl lactose, sialylated Le^a^ and Le^x^. The binding to laminin and H type I was influenced only to a small extent. A clear reduction of bacterial binding to all spotted ligands was observed for the pretreated *H. pylori* with 1 mg/mL FE_90%_.

Therefore, it can be concluded that the okra polysaccharide fraction HBSS interacts with SabA, laminin, and lactoferrin related binding sites. BabA, the major adhesins of *H. pylori* in non-inflamed tissue, is only affected to a minor extent. On the contrary, a preincubation with FE_90%_ leads to a decreased fluorescence intensity for ligands related to BabA, SabA and HpA.

These interactions of okra polysaccharides with bacterial adhesins confirm our findings of the inhibition of *H. pylori* adhesion to gastric epithelial cells under *in vitro* conditions.

Due to the increasing interest in development of antiadhesive compounds against bacterial pathogens the above described experiments are to our knowledge the first study, which indicates that acetylated rhamnogalacturonans can influence bacterial adhesion. This aspect might be interesting for further cytoprotective strategies to use such pectin-like polymers for establishment of preventive strategies against stomach infections, caused by *H. pylori.* The development of antiadhesive compounds that interfere with OMPs and block bacterial adhesion might be an interesting approach for prevention [12]. Most *H. pylori* infections occur during the first two to five years of life [13]. In principle, the development of such antiadhesive compounds toward products for use in food or health products might help to prevent very early infection in children [13]. After antibiotic treatment, some patients experience recurrence of the infection after several months (a problem mainly in developing countries, such as in South America and Asia), and it is possible that these patients might benefit from the use of such compounds in food supplements to be used during and after antibiotic eradication therapy. In this context, translational developments of antiadhesive plant extracts are of interest.

The chemical synthesis and optimization of specific inhibitors of the major adhesins BabA and SabA might be possible, and might be a promising tool for future pharmaceutical and clinical development. For optimized *in silico* definition and chemical synthesis of inhibitors with specific activity toward the active center of these lectin-like proteins, more detailed investigations regarding the molecular and physical characteristics of the adhesins are necessary; specifically, no protein crystal data have been published at this time.

## 3. Experimental Section

If not stated otherwise, all chemicals were purchased from Sigma (Deisenhofen, Germany) and VWR (Darmstadt, Germany) in analytical quality. 3ʹ-Sialyllactose (NeuAcα_2-3_Galβ_1-4_Glc) and fluoresceinisothiocyanate isomer I (FITC) were purchased from Sigma Chemicals (St. Louis, MO, USA). Standard pectins with a defined degree of esterification (DE) were obtained from Roth, Karlsruhe, Germany (DE 8, 38, and 70) and from Fluka, Buchs, Switzerland (DE 60 and 66).

### 3.1. Isolation of Polysaccharides from Immature Okra Fruits

Fresh immature okra fruits were purchased in a local Asian supermarket in Münster, Germany. A voucher species is deposited in the archives of IPBP, University of Münster (No 392 + 393).

A fresh water extract was prepared according to methods described by [1]. In principle, 500 g of the fresh, immature fruits were separated in calyx, pulps and seeds. Three hundred twenty-five grams of pulp was homogenized with 1000 mL Aqua millipore^®^ and centrifuged (12,000× *g*, 4 °C, 60 min). The clear supernatant was dialyzed against Aqua millipore^®^ (cellulose membranes, MWCO 3500 Da, Roth, Karlsruhe, Germany) for 72 h at 4 °C. Five hundred milliliters of the resulting extract (Okra-FE) was subjected directly to ammonium sulfate precipitation. Polymers were precipitated at saturation levels of 30%, 60%, and 90% ammonium sulfate, respectively. The respective pellets were recovered by centrifugation (14,000× *g*, 4 °C, 30 min), dissolved in water, dialyzed, lyophilized and named in the following as FE_30%_, FE_60%_ and FE_90%_. The yields were 0.05%, 0.06%, and 0.14%, respectively, referred to the fresh weight of the pulp material. The remaining supernatant (FE_S_) corresponds to 0.12% of fresh pulps.

### 3.2. Stepwise Extraction of Okra Cell Wall Polysaccharides

For isolation of okra fruit polysaccharides a sequential extraction protocol described by [3] was used. Five hundred grams of fresh immature Okra fruits were separated into seeds, pulp, and calyx. Three hundred sixty grams of resulting pulps were extracted twice with 500 mL of 70% ethanol (*v*/*v*) for 30 min. After filtration, the insoluble residue was washed two times with 500 mL methanol/chloroform (1/1, *v*/*v*) under gentle stirring for 1 h. The solid parts were separated by filtration, extracted with acetone and air dried yielding in 31 g of alcohol insoluble solids (AIS) (9% of fresh pulps). Twenty grams AIS were subjected to a sequential aqueous extraction using 500 mL of the following extractans: Sodium acetate buffer, 0.05 M, pH 5.2, 70 °C, 1 h (hot buffer soluble solids, HBSS); EDTA 0.05 M and sodium acetate 0.05 M in sodium oxalate 0.05 M, pH 5.2, 70 °C, 1 h (chelating agent soluble solids, CHSS); sodium hydroxide 0.05 M supplemented with NaBH_4_ 20 mM, 0 °C, 1 h (diluted alkali soluble solids, DASS); sodium hydroxide 6 M supplemented with NaBH_4_ 20 mM, 0 °C, 1 h (concentrated alkali soluble solids, CASS). Each extraction step was repeated three times. After each extraction the insoluble material was separated by centrifugation (20,000× *g*, 30 min, 4 °C) and subjected to the next extraction step. The clear extracts were combined, dialyzed against Aqua millipore^®^ and lyophilized. The yields obtained for HBSS, CHSS, DASS, and CASS were 63%, 16%, 7%, and 7%, respectively, related to AIS used for extraction.

### 3.3. Carbohydrate Analysis

Polysaccharides were analyzed according to methods described in detail by [14,15,16,17,18,19]. The protein content was determined by Pierce™ BCA Protein Asay Kit (ThermoScientific, Rockford, IL, USA) according to manufactor’s instructions.

Saponification of polymers was performed according to [4]. For removal of methyl and acetyl ester, 30 mg substance were dissolved in 2 mL Aqua millipore^®^ and saponified by adding 3 mL sodium hydroxide 0.1 M. After incubation at 4 °C overnight, the solution was neutralized by 3 mL acetic acid 0.1 M, followed by subsequent dialysis and lyophilisation. Deesterification of polysaccharides yielded in recovery rates of 77%, 72%, and 64% for FE_60%_, FE_90%_, and HBSS, respectively.

### 3.4. Determination of Molecular Weight

HP-SEC was performed on a SECurity GPC system (PSS Polymer Standard Service, Mainz, Germany) consisting of three Suprema^®^ columns from PSS (i.d. 8 mm, particle size; 10 µm 100 Å, 300 mm; 3000 Å, 300 mm; guard column, 50 mm) coupled online to a refractive index detector (Agilent series 1200 RID, Agilent Technologies, Santa Clara, CA, USA), a UV detector (Agilent 1200 Series Variable Wavelength Detector, Agilent Technologies), a viscosimetric detector (PSS SECurity ETA2010) and MALLS detection (PSS SECurity SLD7000 MALLS) equipped with a 5 mW HeNe laser, operating at λ = 632.8 nm. Sodium phosphate buffer (50 mM, pH 7) served as eluent at 0.7 mL/min; Calibration was performed with pullulans (PSS Calibration Kit (1.32, 5.9, 10, 22.8, 47.3, 112, 212, 404, 710 kDa) dn/dc = 0.149 (pullulan) and data analysis by PSS WinGPC Unity V.7.3.0 (PSS Polymer Standard Service, Mainz, Germany).

### 3.5. IR Spectroscopy

IR spectra were recorded with a Shimadzu Prestige 21 FTIR spectrometer (Shimadzu, Tokyo, Japan). For each spectrum 20 scans were recorded with a resolution of 8 cm^−1^.

### 3.6. NMR Spectroscopy

Due to the limited solubility because of the high molecular weight of native polymers in solvents suitable for NMR, fractions HBSS and FE_90%_ were subjected to partial TFA hydrolysis to obtain smaller molecules exhibiting typical structural features. Therefore, 50 mg of HBSS and FE_90%_ were dissolved in 3 mL 0.2 M TFA in a sealed glass tube and incubated at 100 °C for 1 h. TFA was removed by adding 1 mL of methanol 50% (*v*/*v*) with subsequent evaporation to dryness for three to four times. The residues were dissolved in 1 mL 0.15 M NaCl and separated on a Sepharose^®^ CL-6B column using 0.15 M NaCl as eluent at 1 mL/min. Fractions were collected for 2 min and analyzed with resorcinol-sulfuric acid assay for carbohydrate content [20]. Calibration was performed using standard dextrans with molecular weights of 5 kDa, 25 kDa, 50 kDa, and 270 kDa. According to the elution profiles, both hydrolysates were combined yielding one low molecular weight fraction ranging from 5 kDa to 50 kDa and one high molecular weight fraction >50 kDa, named in the following as HBSS-1/FE_90%_-1 for MW >50 kDa and HBSS-2/ FE_90%_-2 for MW 5–50 kDa, respectively. The combined fraction were dialyzed and lyophilized. The recovery yield was 69% and 16% for HBSS-1 and HBSS-2 and 63% and 7% for FE_90%_-1 and FE_90%_-2, respectively.

For NMR, 13 mg of HBSS-1 and FE_90%_-1 were dissolved in 0.8 mL D_2_O (Uvasol^®^, Merck, Darmstadt, Germany) and filtered through prewashed cotton. ^1^H- and ^13^C-NMR measurements were obtained at 600 MHz and 150 MHz, respectively (Agilent VNMRS 600). 2D-NMR data (HSQC) were obtained under the same settings. The NMR spectra were referenced to the C-6 signal of rhamnose (17.20 ppm in ^13^C-NMR, 1.25 ppm in ^1^H-NMR, 26 °C). Data analysis was achieved with MestReNova software, version 10.0.0-14381 (Mestrelab Research S.L., Santiago de Compostela, Spain).

The signals of the anomeric centre were compared to reference standards. To that end, 40 mg of the soluble gum arabic fraction (Caelo, Hilden, Germany) (Defaye and Wong, 1986) and 40 mg of sugar beet arabinan (Westphal, Kuehnel, Schols, Voragen, and Gruppen, 2010) (Südzucker, Obrigheim, Germany) were measured in D_2_O containing 3-(trimethylsilyl)-propionic-2,2,3,3-*d*_4_ acid sodium (TMSP). Signals were referenced to the C-6 of rhamnose at 1.25 ppm in ^1^H- and 17.20 ppm in ^13^C-NMR spectrum.

### 3.7. Cell Culture

Human adherent gastric adenocarcinoma epithelial cells (AGS, ATCC CRL-1730) were kindly provided by W. Beil (Medizinische Hochschule Hannover, Germany). Cells were cultivated as described by [5].

### 3.8. Bacteria and Growth Conditions

*Helicobacter pylori* ATCC 700824 (strain J99, identification for quality control by PCR for vacA and cacA genes) was cultivated for two or three passages to minimize the risk of phase-variable switching of OMP genes. Cultivation was performed according to [21]. Bacteria were grown on tryptic soy agar supplemented with 5% defibrinated sheep blood for 48 h at 37 °C under microaerophilic conditions.

The following clinical isolates of *H. pylori* were used: strain P12 [22], strain 26695 [23] and strain TN2 [24].

### 3.9. FITC-Labeling of Bacteria

According to [25], agar grown bacteria were harvested and resuspended in saline solution (pH 9.0). A bacterial suspension of approximately 1.0 × 10^8^ bacteria (corresponding to an OD_550_ 0.25 in 1:20 dilution) in 1 mL was incubated with 10 µL of a FITC solution (1% in DMSO) for 30 min at 37 °C. Bacteria were recovered by centrifugation (3150× *g*, 5 min) and washed several times with PBS to remove excess of FITC. The resulting pellet was suspended in buffer for further use.

### 3.10. Quantitative Flow Cytometric Adhesion Assay

The adhesion assay was performed as described previously by [2]. In principle, FITC-labeled *H. pylori* were preincubated with test compounds at 37 °C for 2 h. After centrifugation, bacteria were washed three times to remove unbound material. Finally, bacteria were suspended in cell culture media and added to AGS cells, cultivated in 6-well plates for 48 h. The co-incubation of cells and bacteria together with bacteria was performed for 1 h at 37 °C. Unbound bacteria were removed by 2 washing steps. Cells were detached by subsequent trypsinization, resuspended in cell culture media and analyzed by flow cytometry (FACS Calibur, BD, Heidelberg, Germany). Flow cytometry instrument settings: FSC (Detector): E-1 (Voltage), 3.00 (Amp Gain) Lin (Mode); SSC: 352, 1.00, Lin; FL1: 360, 1.00, Log.

### 3.11. Dot Blot Overlay Assay

The dot blot overlay assay was performed as described by [2,13].

### 3.12. Statistics

Results are expressed as mean value (MV) ± standard deviation (SD). After Levene’s test on variance homogeneity, analysis was performed using one-way analysis of variance (one-way ANOVA). If results revealed significant differences between group mean values, then groups were compared using the Student test (2-sided), with *p* < 0.05 considered statistically significant (*) and *p* < 0.01 considered highly statistically significant (**).

## 4. Conclusions

Acetylated rhamnogalacturonans are assessed to be interesting polysaccharides for further investigation and development towards potent antiadhesive pathogen targeting against *H. pylori*. Potential *in vivo* efficacy of such polymers or standardized okra extract has to be proven in future animal infection studies and clinical human investigations.
